# Data on differentially expressed proteins in rock inhibitor-treated human trabecular meshwork cells using SWATH-based proteomics

**DOI:** 10.1016/j.dib.2020.105846

**Published:** 2020-06-12

**Authors:** Sze-Wan Shan, Chi-Wai Do, Thomas Chuen Lam, Hoi-Lam Li, W. Daniel Stamer, Chi-Ho To

**Affiliations:** a Laboratory of Experimental Optometry, Centre for Myopia Research, School of Optometry, the Hong Kong Polytechnic University, Kowloon, Hong Kong, China; b Department of Ophthalmology, Duke University, Durham, NC, United States; c Department of Biomedical Engineering, Duke University,Durham, NC, United States; d The Hong Kong Polytechnic University Shenzhen Research Institute, Shenzhen, China

**Keywords:** Rock inhibitor, Trabecular meshwork, Swath, glaucoma

## Abstract

Rho-associated coiled coil-forming protein kinase (ROCK) inhibitors represent a novel class of anti-glaucoma drugs because of their ocular hypotensive effects. However, the underlying mechanisms responsible for lowering intraocular pressure (IOP) are not completely clear. The protein profile changes in primary human trabecular meshwork (TM) cells after two days treatment with a ROCK inhibitor were studied using label-free SWATH acquisition. These results provided significant data of key protein candidates underlying the effect of ROCK inhibitor. Using the sensitive label-free mass spectrometry approach with data-independent acquisition (SWATH-MS), we established a comprehensive TM proteome library. All raw data generated from IDA and SWATH acquisitions were uploaded and published in the Peptide Atlas public repository (http://www.peptideatlas.org/) for general release (Data ID PASS01254).

Specifications table

**Table:** 

Subject	Ophthalmology
Specific subject area	Cell biology in eyes
Type of data	Fig. Table
How data were acquired	Information-dependent acquisition and data-independent acquisition using Quadrupole Time-of-Flight TripleTOF^Ⓡ^ 6600 mass spectrometer (SCIEX)
Data format	Raw and analysed
Parameters for data collection	Proteins were isolated from primary human trabecular meshwork cells treated with of ROCK inhibitor for two days and untreated controls. Identification and quantification of proteins from the lysate was performed using SWATH mass spectrometry.
Description of data collection	SWATH Mass Spectrometry*;* Quadrupole Time-of-Flight TripleTOF^Ⓡ^ 6600 mass spectrometer (SCIEX); searched against the UniProt database (organism ID: 9606)
data source location	center for myopia research, school of optometry, the Hong Kong polytechnic university, Kowloon, Hong Kong
Data accessibility	All raw data generated from IDA and SWATH acquisitions were available on the Peptide Atlas public repository (http://www.peptideatlas.org/) for general release (Data ID: PASS01254).

## Value of the data

• ROCK inhibitor is a newly developed therapeutic agent for glaucoma, but the underlying mechanism for its ocular hypotensive action is not yet fully understood. This project describes the use of SWATH MS to examine the underlying biological protein changes following ROCK inhibitor treatment of primary human trabecular meshwork (TM) cells. This knowledge may allow more specific anti-glaucoma drugs to be developed.
• Glaucoma patients may benefit from a clearer understanding of the protein changes that are affected by ROCK inhibitor
• Generation of the first published human TM cells ion library for future SWATH-MS study on eye diseases.

## 1 Data description

**Fig. 1:**
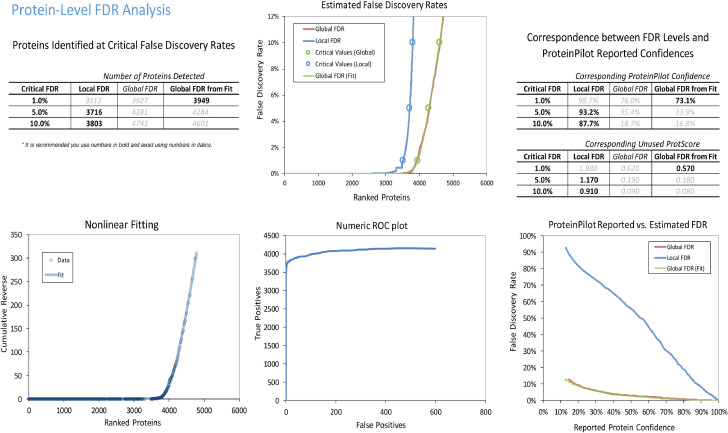
FDR analysis of combined IDA library of human primary trabecular meshwork at protein level generated by Protein Pilot software.

**Fig. 2:**
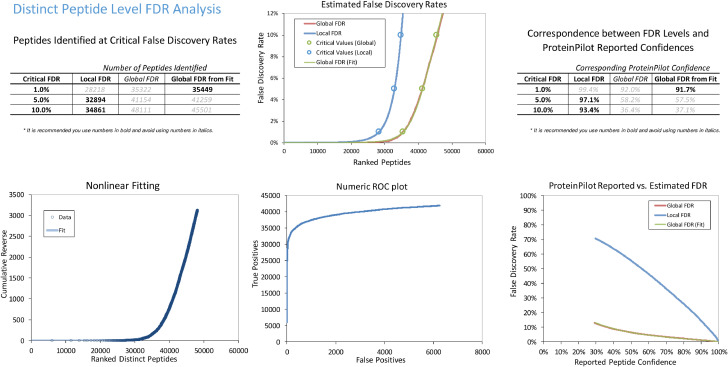
FDR analysis of combined IDA library of human primary trabecular meshwork at peptide level generated by Protein Pilot software.

Proteins were identified using ProteinPilot 5.0 (Sciex, US) software. Fig. 1, Fig. 2 show the protein and peptide FDR analyses, respectively. To generate a reference proteome spectral library of the primary hTM cells for subsequent SWATH analysis, digested peptides were obtained from biological triplicates of each control and treatment group from all cell strains, and hTM proteome was identified by using a discovery based experiment in DDA mode. Protein identification was performed using ProteinPilot 5.0 Software (Sciex, Framingham, MA). A combined IDA library was constructed which consisted of a total of 3949 (at 1% FDR) unique, non-redundant proteins derived from a total of 35,449 (at 1% FDR) distinct peptides. Supplementary Table 1 and supplementary Table 2 shows the full list of identified protein IDs and peptides indentified in the combined ion library respectively.

## 2 Experimental design, materials, and methods

### 2.1. Cell culture

The cells were processed as previously described [1]. Herein, primary human TM cells were obtained from Prof. W. Daniel Stamer at the Department of Ophthalmology, Duke University School of Medicine (Durham, NC) following cell isolation procedures [2], [3], [4]. Three cell strains (i.e. HTM126, HTM129, and HTM136) obtained from three donors, aged 3 months, 30 months, and 75 years, have been characterized previously [2–4]. Cells (at the 3rd or 4th subculture) were plated onto six-well plates (Falcon, Corning, NY) and incubated at 37 °C until confluence. Subsequently, the cells were maintained in Dulbecco's modified Eagle's medium (DMEM, Low glucose; Invitrogen) containing penicillin (100 units/ml), streptomycin (100 mg/ml), glutamine (0.29 mg/ml) (Invitrogen), and 1% fetal bovine serum (FBS) (Invitrogen) for at least seven days before experimentation to keep the TM cells in a resting phase and ready for various assays. Before use the TM cells were incubated in serum-free DMEM overnight. The cells were then treated with a ROCK inhibitor, 1 μM ROCK inhibitor for 48 h. Cells were untreated as control group.

### 2.2. Trypsin digestion of sample preparation prior to lC-MS/MS

Proteins were lysed as previously [1]. After treatment with ROCK inhibitor or vehicle, cells were lysed in a customized lysis buffer containing 7 M urea, 2 M thiourea, 30 mM TRIS, 1% ASB14, 2% CHAPS, and protease inhibitor cocktail (Roche, Mannheim, Germany). Samples were incubated at 4 °C for one hour with sonication, followed by centrifugation at 13,000 × *g* for 20 min. The supernatant was collected and their total protein concentration quantified using the BioRad Protein Assay (BioRad Laboratories, Hercules, CA). Lysates containing 75 µg proteins per sample were reduced with 8 mM dithiothreitol at 37 °C for 45 min. Cysteines were alkylated with 20 mM iodoacetamide at 25 °C in the dark for 30 min. The proteins were precipitated with a four-fold volume of cold acetone after brief vortexing. The mixture was held at −20 °C overnight, followed by centrifugation at 13,000 rpm for 30 min at 4 °C. After removing the supernatant, the pellet was washed with 500 µL of 80% acetone and further centrifuged at 13,000 rpm for 10 min at 4 °C. The protein pellet was air-dried and re-suspended in 1 M urea containing 25 mM ammonium bicarbonate before determining the protein concentration by standard Bradford assay. Trypsin was added to the sample at a 1: 25 w/w ratio (enzyme: protein, ratio in v/v), and the mixture incubated at 37 °C overnight. Trypsin-digested peptides were acidified with a solution of 0.5% formic acid prior to LC-MS/MS analysis.

### 2.3. LC-MS/MS

The settings of LC-MS/MS were as previously used [5–7]. Two technical replicates were performed for each group. Briefly, for data-dependent acquisition and SWATH-MS experiments, a 120-min effective gradient separation was used. Peptides (2 µg) from each sample were first loaded on a trap column (200 μm × 0.5 mm, C18) with loading buffer (0.1% formic acid, 5% acetonitrile in water) at 3 µL/minute rate for 15 min, followed by separation on a nano-LC column (75 µm × 15 cm, ChromXP C18, 3 µm, 120A) using an Ekisgent 415 nano-LC system. The LC separation was performed with a flow rate of 300 nL/min, using mobile phase A (0.1% formic acid, 5% acetonitrile in water) and B (0.1% formic acid in acetonitrile) with the following gradients: 0–1 min, 5% B; 1–121 min, 5–35%B; 121–125 min, 35%B; 125–131 min, 35–80%B; 131–141 min, 80%B; 141–143 min, 80%−5%B; and 143–160 min, 5%B.

All MS data was acquired using a hybrid Quadrupole Time-of-Flight TripleTOF^Ⓡ^ 6600 mass spectrometer (SCIEX, US) with Analyst TF 1.7 software. The ion source was operated according to the following parameters: ISVF = 2300; GS1 = 15; CUR = 30; and IHT = 120. For the Date Dependant Acquisition (DDA) experiment, TOF-MS scan over a mass range 350–1500 *m/z* with 250 ms accumulation time was performed, followed by 100 to 1800 *m/z* MS/MS scanning in a high-sensitivity mode with 80 ms accumulation time of up-to-top 40 ion candidates per cycle. The DDA criteria for the precursor ions included the intensity of ions greater than 125 cps, with a charge state between 2 and 4. The duration of dynamic exclusion was defaulted to 18 s. In SWATH MS-based experiments, the instrument was tuned to specifically allow a quadrupole resolution of 25 Da/mass selection. An isolation width of 25 Da was set in a looped mode over the full mass range (350–1500 *m/z*) scan and 46 overlapping windows were constructed. An accumulation time of 80 ms was set for each MS/MS experiment.

### 2.3. Bioinformatics data analysis

The procedures of the bioinformatics data analysis were as previously described [[5], [6], [7]]. Protein identification was performed using the ProteinPilot 5.0 software (SCIEX, US). The DDA data were searched against the human Uniprot database (ver. 26,095 entries). Searching parameters included trypsin as the enzyme, cysteine alkylated with iodoacetamide (IAA), selections of ‘thorough’ search effort, and using ‘biological modification’. Peptide and protein identification were obtained at 1% false discovery rate (FDR). Only non-redundant proteins were included.

The names of proteins identified as differentially expressed after ROCK inhibitor treatment (IDs) were converted to gene names using the batch id conversion tool in the Uniprot protein online database (http://www.uniprot.org/) for subsequent bioinformatics analysis.

Raw MS data (from both DDA and DIA acquisitions) generated were released to the Peptide Atlas public repository (http://www.peptideatlas.org/) for general access (Data ID PASS01254). For SWATH analysis, a combined peptide spectral library from all information dependent acquisitions (IDAs) of each sample was generated using the identified peptides from all DDA data (a total of six DDA profiling datasets), and the corresponding peptide fragment peaks for each peptide were extracted using the SWATH Acquisition MicroApp 2.0 in PeakView 2.0 software (SCIEX, US). Up to six peptides per protein and up to six fragment transition ions per peptide were used for the peak extraction. Peptides with at least 95% confidence were used, and shared peptides were excluded. The retention time (RT) was aligned by 10 manually selected peptides with high intensity from 20 to 100 min of the run. FDR threshold was set at a default of 1% for filtering false positive quantitation and extracted ion chromatogram (XIC) with a five-min extraction window with 75 ppm fragment mass tolerance. After peak extraction, data were exported into the MarkerView 1.2.1 software (SCIEX, US) for data normalization based on a ‘total area sum’ before statistical analysis. In the data analysis, “significant changes” of differentially expressed proteins were considered if they fulfilled the predefined criteria of: fold change of ≥1.5 or ≤0.67 and *p*-value of ≤0.05 (paired *t*-test), and protein identification at 1% FDR in ≥2 biological replicates.

## Declaration of Competing Interest

The authors declare that they have no known competing financial interests or personal relationships which have, or could be perceived to have, influenced the work reported in this article.

## Appendix Supplementary materials

Image, application 1

Image, application 2
